# High Risk of Heart Tumors after COVID-19

**DOI:** 10.3390/life13102087

**Published:** 2023-10-20

**Authors:** Lubov Mitrofanova, Igor Makarov, Ekaterina Goncharova, Taiana Makarova, Anna Starshinova, Dmitry Kudlay, Evgeny Shlaykhto

**Affiliations:** 1 Almazov National Medical Research Centre, 197341 St. Petersburg, Russia; mitrofanova_lb@almazovcentre.ru (L.M.); doctormakarovia@gmail.com (I.M.); goncharova_ea@almazovcentre.ru (E.G.); makarova_ta@almazovcentre.ru (T.M.); shlyakhto_ev@almazovcentre.ru (E.S.); 2 Department of Pharmacology, I.M. Sechenov First Moscow State Medical University, 119992 Moscow, Russia; d624254@gmail.com; 3 Institute of Immunology, 115478 Moscow, Russia

**Keywords:** SARS-CoV-2, COVID-19-associated tumors, angiopathy, heart tumors, CD68+ macrophages, myxoma

## Abstract

An emergence of evidence suggests that severe COVID-19 is associated with an increased risk of developing breast and gastrointestinal cancers. The aim of this research was to assess the risk of heart tumors development in patients who have had COVID-19. Methods: A comparative analysis of 173 heart tumors was conducted between 2016 and 2023. Immunohistochemical examination with antibodies against spike SARS-CoV-2 was performed on 21 heart tumors: 10 myxomas operated before 2020 (the control group), four cardiac myxomas, one proliferating myxoma, three papillary fibroelastomas, two myxofibrosarcomas, one chondrosarcoma resected in 2022–2023. Immunohistochemical analysis with antibodies against CD34 and CD68 was also conducted on the same 11 Post-COVID period heart tumors. Immunofluorescent examination with a cocktail of antibodies against spike SARS-CoV-2/CD34 and spike SARS-CoV-2/CD68 was performed in 2 cases out of 11 (proliferating myxoma and classic myxoma). Results: A 1.5-fold increase in the number of heart tumors by 2023 was observed, with a statistically significant increase in the number of myxomas. There was no correlation with vaccination, and no significant differences were found between patients from 2016–2019 and 2021–2023 in terms of gender, age, and cardiac rhythm dis-orders. Morphological examination revealed the expression of spike SARS-CoV-2 in tumor cells, endothelial cells, and macrophages in 10 out of 11 heart tumors. Conclusion: The detection of SARS-CoV-2 persistence in endothelium and macrophages as well as in tumor cells of benign and malignant cardiac neoplasms, the increase in the number of these tumors, especially cardiac myxomas, after the pandemic by 2023 may indicate a trend toward an increased risk of cardiac neoplasms in COVID-19 patients, which re-quires further research on this issue and a search for new evidence.

## 1. Introduction

Our knowledge of the coronavirus is constantly being updated. Data analysis from nearly 154,000 people with SARS-CoV-2 gives a dismal projection for long-term cardiovascular outcomes of COVID-19 [1]. After recovery from the acute phase of the disease, many studies within last few years have presented a high risk of different cardiovascular problems, including heart rhythm disturbances, myocarditis, pericarditis, blood clots, strokes, myocardial infarction and heart failure [2]. Furthermore, there was evidence of risks in people who had mild and latent COVID-19 infection.

There is an opinion in the literature that SARS-CoV-2 infection also leads to the development of cardiovascular diseases “de novo” [3]. One of mechanisms of pathogenesis is endotheliitis [4,5]. It is now known that SARS-CoV-2 is able to suppress the immune response to virus entry into the body. Lymphopenia has been described in many patients which is most often characterized by decreased levels in CD4+ and CD8+ T-lymphocytes, which is also a characteristic feature of a number of coronavirus infections [6]. CD4+ T cells specific for SARS-CoV-2 express IFNγ, TNF-ά, IL-2, which indicate the development of a Th1-type cellular response [7]. In addition, direct, long-term damage by cardiomyocyte virus persistence due to the failure of the T-cells immune response are not excluded [8,9]. A decrease in the number of T lymphocytes has been shown in mouse models to be accompanied by the development of intense inflammation in the lungs [10]. At the moment, the pathogenetic mechanisms of myocardial damage are still at the stage of hypotheses [11,12,13].

The current literature pays attention to the potential link between of COVID-19 and long-term cancer risk [14]. Viruses damage host DNA, disrupt apoptosis as well as suppress host immune responses [15]. SARS-CoV-2 contains safe proteins that play important roles in cell cycle progression, metabolism, epigenetics, translation and RNA processing [16]. Some authors believe that long-term COVID-19 can contribute to the development of cancer in recovered patients and accelerate a tumor progression [17]. This theory stems from the increasing body of evidence regarding SARS-CoV-2’s capacity to influence cancer-related pathways, encourage persistent low-grade inflammation and induce tissue damage [18]. Mendelian randomization to investigate the causal relationship between COVID-19 and 33 different types of carcinomas in the European population revealed an increased risk of HER2-positive breast cancer, esophageal, gastric and colon cancer with a genetic predisposition to severe coronavirus infection [19]. Aim of the study: To assess the risk of developing heart tumors in patients who have had COVID-19.

## 2. Materials and Methods

A retrospective cohort analysis of surgical, biopsy and autopsy archival material of the pathoanatomical department patients who are different ages in Almazov National Medical Research Centre was carried out from 2016 to 2023 inclusive. Patients with operated heart tumors ranged from 27 to 83 years old, and mean age was 60 ± 12 years. According to the study design patients with tumors of the heart were added. There were no exclusion criteria. 

A total of 173 heart tumors were analyzed. Among them, there were 161 tumors of the heart, using operating material. Among them, were 95 females and 66 males. Endomyocardial biopsy (EMB) was performed in 12 patients. Among them, there were 5 female and 7 males, with ages ranging from one day old (newborn with rhabdomyoma) to 53 years old. The diagnosis of a particular tumor was made on the basis of histological examination using hematoxylin and eosin staining and used van Gieson’s stain, and immunohistochemical examination using antibodies to vimentin, CD34, CD31, CD45, CD20, CD5, CD3, CD38, CD56, CD99, panCK, EMA, MUC4, D2-40, WT1, calretinin, mesotelin, melanA, S100, SMA, desmin, MyoD1, myogenin, ERG, FLI1, p53, ALK. Determination of ALK gene rearrangement and MDM2 gene amplification were directed by fluorescence in situ hybridization (FISH).

The medical histories of all patients with cardiac tumors were reviewed, taking into account data on cardiac rhythm abnormalities, heart failure, history of PCR-confirmed coronavirus infection, and vaccination. All patients underwent transthoracic and/or transesophageal echocardiographic evaluation and magnetic resonance imaging in cases of suspected malignancy. Only one case out of 173 was not diagnosed as a tumor (chondrosarcoma). Based on the data of the two methods applied, the clinical diagnosis was restrictive cardiomyopathy and atrial calcification.

The immunohistochemical study with antibodies to spike SARS-CoV-2 (rabbit polyclonal antibody; GeneTex, Hsinchu City, Taiwan; dilution 1:100) was performed in 21 cardiac tumors: 10 mixomah operated until 2020 (control group); 4 myxomas, 1 proliferating myxoma, 3 papillary fibroelastomas, 2 myxofibrosarcomas, 1 chondrosarcoma, resected in 2022-2023. In the same 11 post-COVID heart tumors, an immunohistochemical analysis was performed with antibodies to CD34 (mouse monoclonal antibody, clone QBEnd/10; DAKO, Great Britain; (dilution 1:50) and CD68 (mouse monoclonal antibody, clone PG-M1; DAKO, Carpinteria, CA, THE USA; (dilution 1:25). In 2 cases out of 11 (proliferating myxoma and classic myxoma), an immunofluorescent study was performed with an antibody cocktail to spike SARS-CoV-2/CD34 and spike SARS-CoV-2/CD68. AlexaFluor 647® was used as the first secondary antibody (Abcam, Great Britain), giving red fluorescence, and as second secondary antibodies–AlexaFluor 488^®^ (Abcam, Cambridge, UK), giving green fluorescence. After washing, the samples were counterstained with DAPI (AppliChem, Darmstadt, Germany) and in DAKO environment they were closed. The resulting micropreparations were examined on a Leica DM6000B fluorescent microscope.

The statistical analysis of the obtained data was conducted using Python programming language (3.6 version) libraries such as NumPy, Scipy, Matplotlib, Seaborn and Pandas. For normally distributed data, we represented the results as mean values with confidence intervals, while for non-normally distributed variables, we used medians along with the 25th and 75th percentile values. In comparing scores between groups, we applied various methods, including the Student’s t-test, permutation test or non-parametric Mann–Whitney U-test. Statistical significance was established when *p* < 0.05. We employed the Fisher’s exact test for comparing data frequencies between groups. To identify distribution trends, we use linear regression models, as well as quadratic and cubic approximations.

## 3. Results

The results of surgical material for the specified period of time with a comparison of detected heart tumors before and after COVID-19 is presented in Table 1.

**Figure 1:**
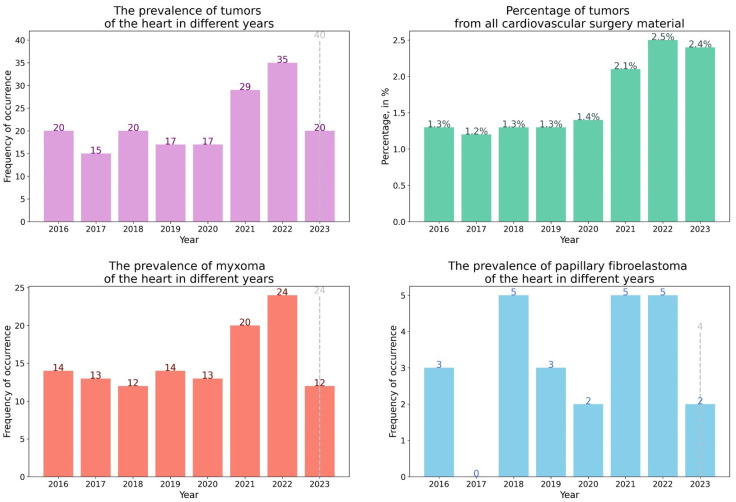
The prevalence of diagnosed heart tumors by years. The dotted line shows the expected number of tumors by the end of 2023.

**Figure 2:**
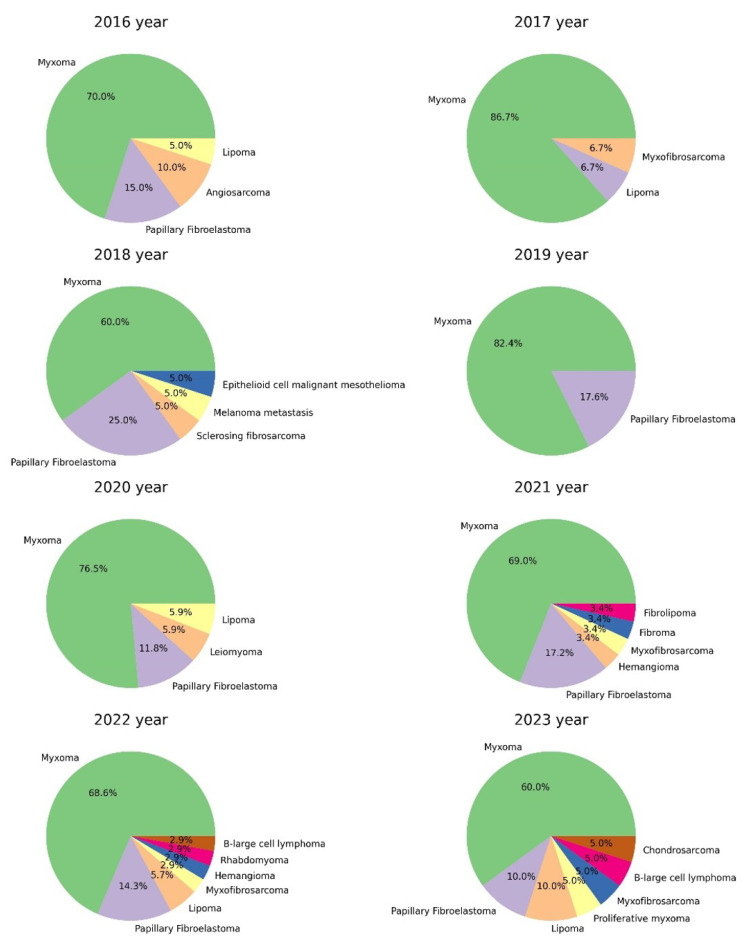
Histograms of the proportion and absolute number of heart tumors in the material of cardiovascular surgery and endomyocardial biopsies from 2016 to 2023 (%).

**Figure 3:**
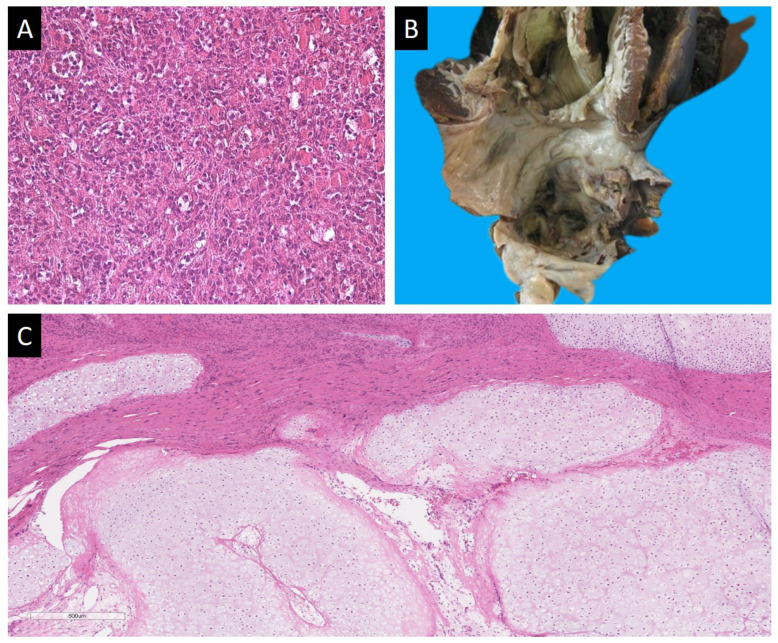
(**A**) Proliferating myxoma of the heart; H&E, ×200. (**B**) Chondrosarcoma of the left atrium. (**C**) Intramural growth of chondrosarcoma in the left atrium; H&E, ×100.

As we presented in Table 1, in 2022, the number of detected heart tumors significantly increased according to the histological examination of surgical material. For 6 months of 2023, 20 heart tumors were detected, which accounted for 0.25% of surgical material, 2.4% of cardiovascular surgery material, 5% of EMB and 0.8% of sectional cases. Among them were 12 myxomas, 1 proliferating myxoma, 2 papillary fibroelastomas, 1 left atrial myxofibrosarcoma, 1 secondary large B-cell lymphoma, 2 lipomas, 1 left atrial chondrosarcoma with spread to the right atrium, interventricular septum and left ventricle (a sectional case) (Table 2) (Figure 1, Figure 2 and Figure 3). The dynamic of various types heart tumors detection in the period from 2016 to 2023 (6 months) is shown in the Figure 1 and Figure 2.

Almost all types of arrhythmias were observed in 64.5% of cases in patiets with heart tumors, but more often there was atrial arrhythmias. Only in 31% of cases patients did not have chronic heart failure. Two out of three patients with EMB-diagnosed myxofibrosarcoma had a successful heart transplant, while chondrosarcoma was diagnosed only at autopsy. The patient’s heart was 17 × 17 × 12 cm in size, weighing 614 g, the walls of the left atrium were of stony density, with uneven relief, up to 0.5 cm thick; the walls of the left and right ventricles were thickened (1.6–1.9 and 0.4–0.9 cm, respectively) due to the white, dense endocardium. Histological examination revealed chondrosarcoma with calcification of the left atrium, with germination of the interatrial and interventricular septum, the wall of the right atrium and left ventricle, “creeping” along the ventricular endocardium. The classical structure of the tumor was observed in the atria, while in the interventricular septum and ventricles, the histoarchitectonics of the tumor corresponded to dedifferentiated chondrosarcoma. Metastases of dedifferentiated chondrosarcoma were found in the liver.

There were 29 patients with heart tumors in 2021. Of these, 14 had COVID-19 and 9 were vaccinated with Sputnik-V. All patients, who were operated or subjected to EMB in 2022, had COVID-19, and 18 out of 35 were vaccinated (13—Sputnik-V, 3—Sputnik light vaccine, 2—Epivaccorona vaccine). All patients, who underwent surgery or EMB in 2023, had COVID-19 and 8 out of 20 were revaccinated in 2022 (5—Sputnik-V, 3—Sputnik light vaccine). None of the patients with cardiac tumors were hospitalized with a severe course of acute COVID-19, but each had symptoms of post-acute sequelae of SARS-CoV-2 infection, including dyspnea, chest pain and heaviness, cognitive impairment, sleep disturbance, headache, fatigue, anosmia and others.

**Figure 4:**
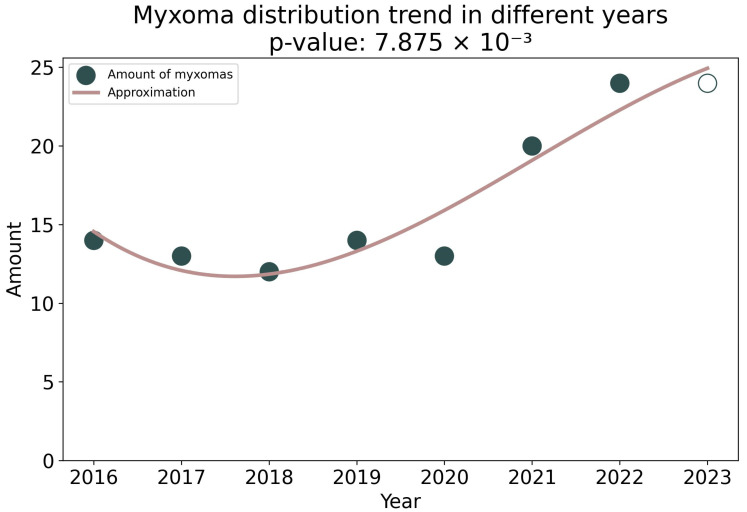
Cubic approximation of the amount of myxoma from 2016 to 2023. A trend toward an increase in the amount of myxoma is shown. The unfilled circle shows the expected number of mixomas in 2023.

Statistical analysis did not reveal significant differences in gender, age, or rhythm disturbances in patients with tumors of the pre-COVID and post-COVID periods. There was no correlation between the type of heart tumor and past COVID-19, as well as vaccination. However, the number of cardiac tumors in the post-COVID period increased by 1.5 times. A trend toward an increase in the number of myxomas from 2016 to 2023 was revealed at *p* = 7.875 × 10^−3^ (Figure 4).

**Figure 5:**
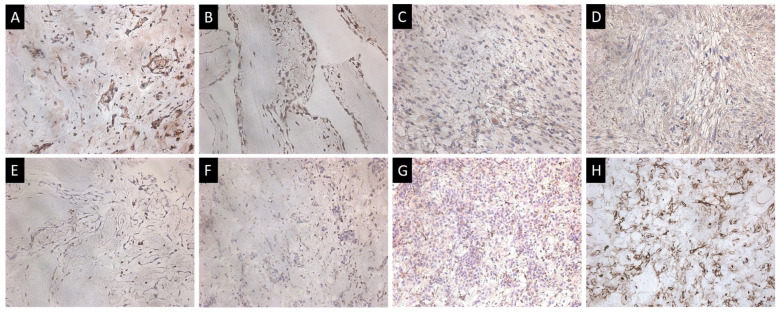
Immunohistochemical study of heart tumors. (**A**–**D**) Expression of spike ARS-CoV-2 in myxoma, papillary fibroelastoma, myxofibrosarcoma in the dedifferentiated zone of Grade 3 chondrosarcoma. (**E**–**G**) CD68+ macrophages in papillary fibroelastoma, classical myxoma and proliferating myxoma. (**H**) CD34+ cells in proliferating myxoma; ×200.

Immunohistochemical study in the control group did not reveal the expression of spike SARS-CoV-2 Spike protein, while the virus protein was detected in tumor cells and macrophages in 4 mixomes (including the proliferating one) (Table 3, Figure 5).

**Figure 6:**
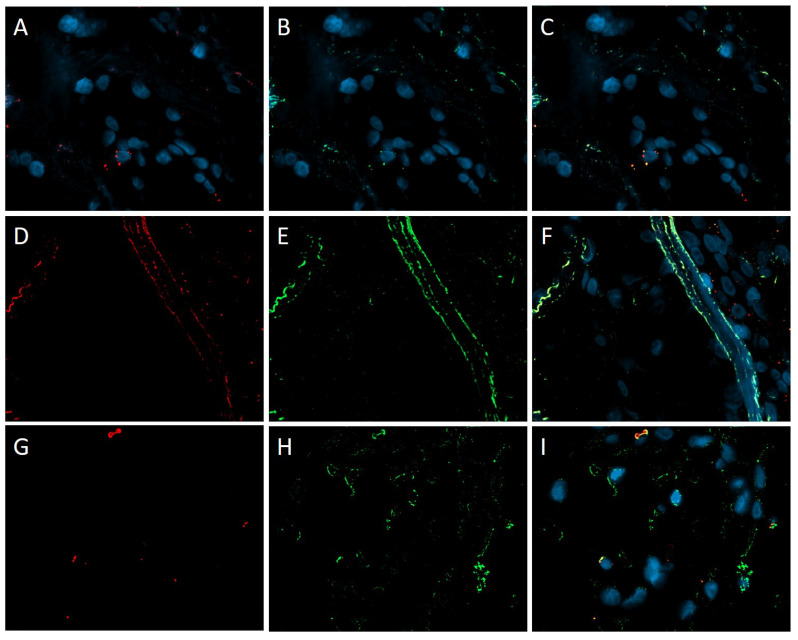
Immunofluorescence microscopy of cardiac tumors. (**A**–**F**) spike SARS-CoV-2/CD34 in tumor cells and vascular endothelium of proliferating myxoma. (**G**–**I**) spike SARS-CoV-2/CD68 macrophages of classical cardiac myxoma. CD34 and CD68—red fluorescence, spike SARS-CoV-2—green fluorescence, DAPI in cell nuclei—blue fluorescence, co-expression of spike SARS-CoV-2/CD34 and spike SARS-CoV-2/CD68—yellow and orange fluorescence; ×630.

It was determined in the cells of the endothelial CD34+ lining of all papillary fibroelastoma. Expression of spike SARS-CoV-2 was also observed in atypical cells of myxofibrosarcoma and chondrosarcoma, as well as liver metastasis of the latter. Immunofluorescence microscopy confirmed the expression of the virus in tumor cells and macrophages of proliferating and classical mixomas, and also showed the fluorescence of spike SARS-CoV-2 Spike protein in vessels (Figure 6).

## 4. Discussion

According to the literature data, cytokines IL-1, IL-6, IL-8 and TNF-α participate in immune response in patients with COVID-19. As we know, they provoked tumor genesis [20]. Additionally, T-cell depletion, including JAK-STAT, MAPK and NF-kB, is associated with COVID-19, and activated oncogenic pathways. It potentially increases cancer risk [21]. The depletion of angiotensin-converting enzyme 2 (ACE2) induced by viruses and inflammation due to hypoxia can lead to oxidative stress and promote malignant transformation. DNA damage and subsequent carcinogenesis led to chronic inflammation and oxidative stress [22,23,24]. It is known that COVID-19 causes multiple organ damage [25], and extensive tissue damage is an oncogenic factor. Research on the closely related SARS coronavirus suggests a potential link between SARS-CoV-2 and cellular transformation. SARS-CoV-2 nonstructural protein 3 (Nsp3) is involved in the degradation of the tumor suppressor protein p53 [26]. Moreover, the S2 subunit of SARS-CoV-2, p53 and BRCA1/2 interacted with each other [27]. Gatekeepers and cellular guardian genes loss can guide to genomic instability and aberrant cell growth, as well as cellular disorders [28,29,30]. On the flip side, COVID-19 and cancer share several similarities, including an inadequate T-cell response. They also have commonalities in terms of antigenic stimulation triggered by damage-associated molecular pattern (DAMP) and pathogen-associated molecular pattern (PAMP) molecules, which occur in both cancer and infectious diseases [31]. DAMP and PAMP play pivotal roles in inflammation, culminating in the release of various cytokines, elevated levels of reactive oxygen and nitrogen species, tissue damage and apoptosis. Moreover, the hypoxic microenvironment induces the production of lysyl oxidase (LOX), thereby promoting tumor cell invasion and facilitating their migration and metastasis [32].

Bouhaddou M. et al. demonstrated alterations in the phosphorylation status of host and virus proteins following SARS-CoV-2 infection. They observed phosphorylation changes in 12% of interacting host proteins and pinpointed the responsible kinases that regulate cell shape and activate the p38/MAPK pathway [33]. While the majority of infected cells undergo apoptosis, some manage to survive after the virus is eliminated. It remains unclear whether the phosphorylation status of host proteins is restored to its original state or if persistent aberrant phosphorylation could eventually lead to oncogenesis. Cancer development seldom arises from isolated events; it typically results from the accumulation of mutagenic events over an extended period. In conjunction with other carcinogenic factors, COVID-19 may predispose the body to tumor development and expedite its progression.

Several hypotheses have been put forward regarding the integration of SARS-CoV-2 into the human genome [34,35]. The researchers studied the ability of SARS-CoV-2, as a positive-strand RNA virus, to integrate into the human genome after reverse transcription. Transcription of integrated sequences is the logical proof of repeating PCR-positive tests. However, a number of other researchers deny the integration of SARS-CoV-2 into the human genome [36,37,38].

Although the ability of SARS-CoV-2 to integrate into the host genome is still disputed, and it is not recognized as a traditional oncovirus, it is possible that the virus creates an oncogenic environment by stimulating host immunity. Thus, great attention must be paid to understanding and suppressing internal immune dissonance.

In our study, we found a clear increase in the number of not only myxomas, but also myxofibrosarcomas, which are extremely rare for the heart. The histogenesis of myxoma is poorly understood, but conventional wisdom favors an origin from primitive pluripotent mesenchymal cells. Genes encoding cardiac progenitor markers can be reactivated and expressed in cardiac myxoma cells, resulting in differentiation along endothelial/endocardial lineages. Previously, myxomas were thought to arise from Pritchard’s masses, microscopic endocardial/endothelial structures lined with “puffy” endothelial cells located in the oval fossa [39]. Immunohistochemically, they express CD31, CD34, CD56, FVIIIAg, protein S-100, calretinin, vimentin, desmin, smooth muscle myosin, α1-antitrypsin and alpha-1-antichymotrypsin [40]. Moreover, some of these proteins express normal endothelium. Myxofibrosarcoma, in turn, belongs to the group of intimal sarcomas [41].

For the first time in all the years of work of our center, we diagnosed chondrosarcoma of the left atrium. It is assumed that the tumor arises from multipotent mesenchymal stem cells that undergo malignant cartilage differentiation [42,43]. The tumor often originates from the endocardium, grows into the atrial or ventricular cavity, infiltration progresses through the myocardial wall and spreads to the pericardium and mediastinal structures [44]. In our case, the tumor in the atria grew either intramurally or spread along the endocardium, while in the ventricles, it grew only in the endocardium, mimicking restrictive cardiomyopathy.

Immunohistochemical study found SARS-CoV-2 expression in 10 out of 11 tumors. Moreover, this study, together with immunofluorescence microscopy, revealed SARS-CoV-2 Spike protein in tumor cells, macrophages, vascular endotheliocytes and the endothelial lining of papillary fibroelastoma.

Thus, heart tumors, the number of which increased after the pandemic with the appearance of extremely rare sarcomas, in our opinion, are clearly associated with endothelial dysfunction.

In turn, endothelial cell dysfunction is a characteristic complication of COVID-19 [45]. At the pathological level, endotheliitis is the end result regardless of direct viral infection or indirect effects independent of infection. In addition, it is assumed that SARS-CoV-2 can persist in the endothelium [46,47,48], which once again confirmed our study. Although the role and clinical significance of the endothelium in protracted COVID requires further elucidation, the evidence for its emergence as a potential key player is compelling.

The discovery of SARS-CoV-2 Spike protein in tumor macrophages also came as no surprise to us. Bearse M et al. described an increased number of macrophages in the myocardium during acute coronavirus infection [49]. Other authors write about the persistence of the virus in these cells in the post-acute sequelae of COVID-19 [50,51]. And some researchers prove that macrophages and monocytes can serve as carriers of SARS-CoV-2 [52].

We know that oncogenic viruses cause mutations and cell transformation as a result of viral infection due to modulation of the cell cycle. SARS-CoV-2 infection probably blocks the cell cycle, leading to the activation of apoptosis mechanisms [53]. For persistence, viruses must avoid elimination by the host immune response and elimination of all infected cells while retaining their genomes in some infected cells. This is usually accompanied by low levels of viral replication, and a prolonged virus–host interaction occurs [54]. A distinction is made between productive and non-productive persistence. In productive persistence, virions are detected continuously or periodically, whereas in non-productive persistence, viruses are not produced even though the viral genome remains intracellular and there may be the expression of some viral proteins that activate innate immune receptors, supporting the inflammatory process [55]. All known human oncoviruses are detected in some form of tumor zone. They can persist as chronic infections or persist as nuclear episomes or integrated genomes in virus-associated tumor cells. In chronic infection, the virus does not infect the cancer cell, but its long-term persistence causes inflammation that promotes the emergence of cancer cells, as appears to be the case with SARS-CoV-2 [56]. Discussing viral oncogenic mechanisms encompasses various factors, such as the prevention of apoptosis, alterations in host metabolism, adjustments to the cellular microenvironment, weakening of host immune regulation, modifications in transcription and changes in the epigenome. Many authors believe that the hallmark of SARS-CoV-2 is metabolic reprogramming [57,58,59]. At the same time, the oncolytic effect of the virus on lymphoproliferative diseases is known to occur, including natural killer cell lymphoma and Hodgkin’s lymphoma [60]. The potential causal relationship between SARS-CoV-2 and cancer, as well as the virus’s actual contribution to oncogenesis, remains an unresolved inquiry. This uncertainty arises from instances of oncoviruses reactivating post-COVID-19 and the puzzling reactions of certain tumors to immune modulation [61]. 

Simultaneously, the extended persistence of SARS-CoV-2 within the cardiac endothelium might elevate the likelihood of tumor formation. The virus can cause damage to the endothelium, causing endotheliitis and disturbances in the cellular mechanisms of regulation of cell growth and division; for example, due to the inhibition of oncosuppressors and metabolic reprogramming. In turn, this can contribute to endotheliocyte dysplasia, the emergence of atypical cells and their subsequent development into a tumor. In addition, the inflammatory processes caused by SARS-CoV-2 may enhance the environmental conditions for the development of tumors [62,63].

The results of our study contradict the data of Li J. et al. [19], who concluded that the number of heart tumors in Europe did not increase, but the authors analyzed the data only for 2021, and we for 2021–2023. It cannot be ruled out that an increase in the number of visits with cardiovascular problems after an infection has led to the early detection of heart tumors. Obviously, the so-called cumulative effect cannot be ruled out, when patients could not reach a specialized center due to a pandemic, but all cardiosurgical departments of our center were fully operational in 2021–2023.

On the other hand, close attention to patients with COVID-19, thrombosis and thromboembolism of different localizations not only during acute infection, but also during post-acute sequelae of SARS-CoV-2 [64] allowed for the identification of cardiac tumors in some of them. In particular, a right atrial myxoma with massive thrombus 4 months after coronavirus infection [65] and an ischemic stroke in a patient with left atrial myxoma 2 months after COVID-19 [66] were reported. SARS-CoV-2 infection has been activated the blood coagulation cascade in addition to endothelial dysfunction and hyperinflammation caused by cytokine storm. The action of these factors may persist even after the acute phase of infection [67].

According to Kell DB et al. [68], the etiology of post-acute sequelae of COVID-19 at the molecular level is due to the appearance of aberrant amyloid fibrin microclots. The formation of it is triggered by the adhesion protein SARS-CoV-2. Microclots after COVID-19 are also thought to be a novel antigen that may provoke an autoimmune response. It is formation may explain some of the symptoms of post-acute sequelae of COVID-19. The SARS-CoV-2 virus protein has been provided to fibrinogen in the normal clotting cascade and causes the formation of abnormal clots with an increased proinflammatory response, leading to blood clots in various parts of the body. At the same time, concerns about hypercoagulability in COVID-19 may lead to misdiagnosis of cardiovascular disease in this infection. A case is described in which the patient’s symptoms and the results of transesophageal echocardiography were considered as COVID-19-induced thrombosis on the prosthetic valve in a patient with recurrent dedifferentiated liposarcoma of the heart [69].

Future research is of utmost importance for delving into shared molecular pathways and potential connections between COVID-19 and the advancement of tumors. It is crucial to determine whether the virus may indeed be regarded as an etiological factor in tumor initiation [70]. Regardless, cardiologists should maintain a vigilant focus on patients exhibiting symptoms of post-acute sequelae of SARS-CoV-2.

## 5. Conclusions

The detection of SARS-CoV-2 persistence in endothelium and macrophages as well as in tumor cells of benign and malignant cardiac neoplasms raises concerns. The observed rise in the number of these tumors, especially cardiac myxomas, after the pandemic in 2023, may indicate a trend toward an increased risk of cardiac neoplasms in COVID-19 patients, which requires further research into this issue and a search for new evidence.

## 6. Limitation of the Study

A limited number of patients with cardiac tumors in post-acute sequelae of SARS-CoV-2 has been studied. A further study on a larger sample is planned.

## Figures and Tables

**Table 1:** Results of surgical material with morphological data for the period from 2016 to 2022.

Year of the Study	The Total Number of Surgical Material Morphological Examinations (*n*)	Number of Heart Tumors Detections (*n*/%)	Characteristics of Heart Tumors
2016	8333	17 (0.20)	14 cardiac myxomas and 3 papillary fibroelastomas
2017	8235	14 (0.17)	13 cardiac myxomas and 1 lipoma
2018	10,526	20 (0.19)	12 cardiac myxomas, 5 papillary fibroelastomas, 1 sclerosing epithelioid fibrosarcoma of the right atrium, 1 epithelioid cell malignant mesothelioma, 1 melanoma metastasis.
2019	10,011	17 (0.17)	14 cardiac myxomas and 3 papillary fibroelastomas
2020	9411	16 (0.17)	13 cardiac myxomas, 2 papillary fibroelastomas, 1 leiomyoma
2021	12,707	28 (0.22)	20 cardiac myxomas, 5 papillary fibroelastomas, 1 cavernous hemangioma, 1 fibrolipoma, 1 fibroepithelial polyp
2022	12,918	31 (0.24)	24 cardiac myxomas, 5 papillary fibroelastomas, 1 cavernous hemangioma, 1 lipoma

**Table 2:** Detection of heart tumors by endomyocardial biopsies from 2016 to 2023.

Year	2016	2017	2018	2019	2020	2021	2022	2023
Tumors	Angiosarcoma—2 Infiltrating lipoma	Myxofibrosarcoma	None	None	Lipoma	Myxofibrosarcoma	Myxofibrosarcoma B-large cell primary lymphoma Lipoma Rhabdomyoma	Myxofibrosarcoma B-large cell secondary lymphoma
Total:	3	1	0	0	1	1	4	2

**Table 3:** Proportion of cells expressing spike SARS-CoV-2 Spike protein and macrophages in different cardiac tumors.

№	Year	Diagnosis	Percentage of Tumor Cells Expressing SARS-CoV-2 Spike Protein	Percentage of Cells in a Tumor Expressing CD68
1	2022	Papillary fibroelastoma	95	30
2	2022	Papillary fibroelastoma	98	48
3	2023	Papillary fibroelastoma	98	7
4	2022	Cardic Myxoma	98	48
5	2022	Cardic Myxoma	0	75
6	2022	Cardic Myxoma	93	38
7	2023	Cardic Myxoma	42	49
8	2023	Proliferating myxoma	72	77
9	2023	Chondrosarcoma	87	61
10	2022	Myxofibrosarcoma	87	32
11	2022	Myxofibrosarcoma by EMB	7	1

## Data Availability

Should you require further explanations or need additional details, please feel free to contact us via email at mitrofanova_lb@almazovcentre.ru.
